# Biosensor in smart food traceability system for food safety and security

**DOI:** 10.1080/21655979.2024.2310908

**Published:** 2024-02-01

**Authors:** Catarina Meliana, Jingjing Liu, Pau Loke Show, Sze Shin Low

**Affiliations:** a Nottingham Ningbo China Beacons of Excellence Research and Innovation Institute, University of Nottingham Ningbo China, Ningbo, Zhejiang Province, China; b College of Automation Engineering, Northeast Electric Power University, Jilin, Jilin Province, China; c Department of Chemical Engineering, Khalifa University, Abu Dhabi, Abu Dhabi Municipality, United Arab Emirates; d Department of Chemical and Environmental Engineering, Faculty of Science and Engineering, University of Nottingham Malaysia, Semenyih, Selangor Darul Ehsan, Malaysia

**Keywords:** Biosensors, traceability tools, food safety, food analysis, smart packaging, food contaminants

## Abstract

The burden of food contamination and food wastage has significantly contributed to the increased prevalence of foodborne disease and food insecurity all over the world. Due to this, there is an urgent need to develop a smarter food traceability system. Recent advancements in biosensors that are easy-to-use, rapid yet selective, sensitive, and cost-effective have shown great promise to meet the critical demand for onsite and immediate diagnosis and treatment of food safety and quality control (i.e. point-of-care technology). This review article focuses on the recent development of different biosensors for food safety and quality monitoring. In general, the application of biosensors in agriculture (i.e. pre-harvest stage) for early detection and routine control of plant infections or stress is discussed. Afterward, a more detailed advancement of biosensors in the past five years within the food supply chain (i.e. post-harvest stage) to detect different types of food contaminants and smart food packaging is highlighted. A section that discusses perspectives for the development of biosensors in the future is also mentioned.

## Graphical abstract

**Figure:**
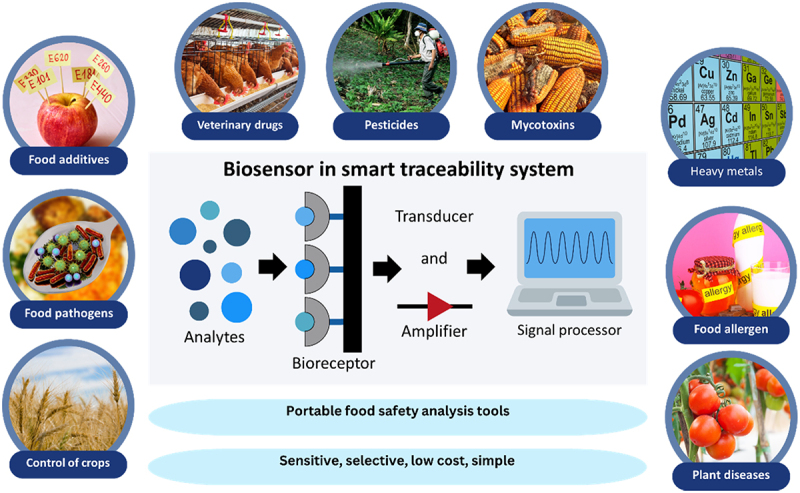

## 1. Introduction

Ensuring access to sufficient amount of safe and nutritious food yet environmentally friendly have been a growing attention since the last decades. It is notorious that the increasing prevalence of foodborne diseases has contributed significantly to the global burden of disease and mortality. This results in public health problems as well as economic and social concerns worldwide. According to the World Health Organization (WHO), there are 600 million (i.e. about 1 in 10 people in the world) cases of people becoming ill after eating contaminated food. This number contributes to the global deaths of 420,000 and the loss of 33 million healthy years of life in 2010 [1]. Data from the annual report on food security and nutrition further stated that nearly 8.9% of the total population, or 690 million people, in the world are hungry, although there is sufficient food to feed the world’s population [2,3].

The burden of foodborne diseases and food insecurity as part of food sustainability issues have influenced both developed and developing countries. However, the highest burden occurs in low- and middle-income countries (i.e. developing countries) that have a high level of poverty and pollution. Rapid urbanization, changes in consumer habits, globalization, and climate change have been known to underpin greater challenges to ensuring food safety and security [4]. According to the global estimates, there are 31 foodborne hazards causing 32 diseases, with the most prominent cases being caused by bacteria, viruses, parasites, or chemical substances through contaminated food [4]. Meanwhile, food waste and loss are strongly linked to food insecurity and a high carbon footprint [5].

Contamination of food, along with food loss and waste, may occur at any stage throughout the food supply chain (i.e. the process from farm to fork, including manufacturing, packaging, distribution, storing, and further processing or cooking for consumption). This is because the process inherently deals with the uncertainty of safety and quality aspects [6,7]. Due to this, traceability across the supply chain must be maintained and continually developed by all sectors (i.e. government, researchers, food industry, and consumers). For instance, government should strengthen the requirement of legislation and certification in the food industry. Meanwhile scientific and industry sectors should cooperate in developing better food traceability system for ensuring food safety and quality. Consumers, at the end, should demanding more food information.

Smart food traceability has been known to significantly help overcome the global challenges related to food omics (i.e. the food fingerprint, which covers the nutritional values, quality, and authenticity of foods, as well as their safety and security) [8]. Biosensors have been known to be reusable and able to replace conventional analytical techniques by giving rapid, accurate, reliable, and multiple analyses [9,10]. Many significant advancements of biosensors for food safety and analysis have been explored, including portable detections of foodborne disease agents in contaminated food. The main principle of detection by biosensors is the combination of a bioreceptor (i.e. biological recognition element) with a transducer (i.e. sensing element), generating a measurable signal proportional to the concentration of analytes. Different types of biosensors have been discovered based on the bioreceptor type (e.g. enzymes, antibodies, microbes, etc.), yet the significance is usually based on the interaction with analytes (i.e. the need to be highly specific) [9]. Alternately, the most common type of biosensor based on its transducer type is electrochemical, while others include optical and mass-sensitive biosensors [11].

Although there are many review articles discussing recent developments of biosensor in food system [12–17], yet it is still limited to found one that discuss applications of biosensor in a whole complex system of food supply chain. The presence review article aims to combine previous studies of biosensors in food safety and analysis from pre-harvest to post-harvest stage. Highlights on the advantages of different biosensors developed within the past 5 years in correlation with smart traceability system are discussed. In general, there are three sections in this review. In the first section, general concepts and the development of food traceability systems and application of biosensors as traceability tools from pre-harvest to post-harvest are discussed. In the second section, information regarding mechanisms and applications of biosensors in food safety and security is mentioned. Lastly, the challenges and future perspectives of recent developments are mentioned.

## 2. Food traceability and biosensor

### 2.1. Food traceability

The International Organization for Standardization (ISO) and Codex Alimentarius Commission (CAC) define *food traceability* as the ability to follow or track the movement or progress of a product (i.e. feed or food) through the food chain, including production, processing, and distribution [18]. Another extent to which the definition of food traceability relates to assurance of food safety is made by the American Production and Inventory Control Society (APICS) [18]. The drivers or motivating factors determining the necessity of food traceability often differ depending on the specific information needed along the supply chain. A review by Islam & Cullen [19] classified the drivers into five categories, including (1) legislation and certification, (2) safety and quality, (3) customer satisfaction, (4) sustainability, and (5) value and efficiency.

It is notable that the urgency for assurance and transparency of food safety within the food supply chain underlined those five driver categories. For instance, certain legislation and certification of a reliable traceability system are required to ensure fair practices in food trade and facilitate the free movement of safe food products within the region [20]. Study by [21] and [22] to assess consumer preferences and willingness to pay for traceable food further proves the statement that food traceability can provide customer satisfaction. The rationale behind this is the significant number of potentially substantial disruptions (e.g. food pathogens, climate, etc.) during the supply chain and the occurrence of food fraud for economic gain that results in unsafe and unsuitable food for consumption [23]. As a result, more people are becoming more knowledgeable about food and demand food credibility or food supply chain transparency.

**Figure 1.**
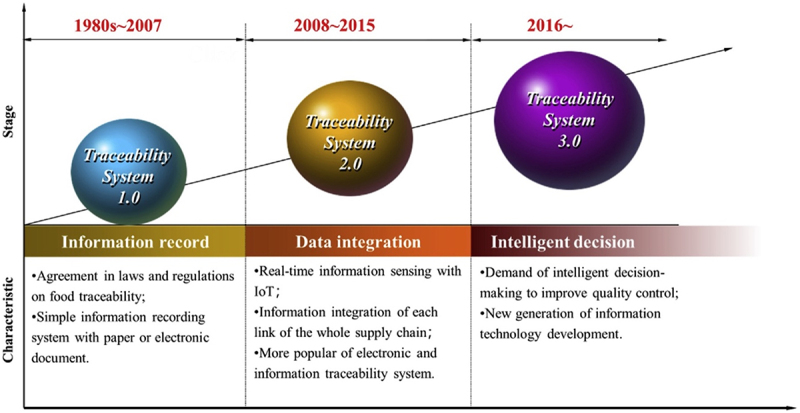
Development stages of food traceability system. Reprinted from [18] with permission from Elsevier.

Food traceability enables whole process monitoring of the uncertainty and complexity of the food supply chain. Thus, ensuring food safety and quality that prevent food waste and the possibility of food contamination, causing foodborne illness [24]. With rapid technological advancements, traceability systems have progressed to a smarter or more intelligent system (Figure 1). The main principles of smart food traceability are to leverage portable sensors and indicators to collect more comprehensive, traceable, and timely data about food products. The leading group of technologies developed includes portable detection devices, smart indicators and sensors incorporated into food packages, data-assisted whole genome sequencing, and other new digital technologies (e.g. Internet-of-Things (IoT) and cloud computing) [8]. Table 1 summarizes the advantages and disadvantages of current portable technologies.

**Table 1.** Advantages and disadvantages of portable traceability technologies.

Technology	Advantage	Disadvantage	Ref
Vibrational spectroscopy	Simple, low cost, portable, no or minimum sample preparation, robust, rapid	Unrealistic measurement due to low resolution, narrow wave number, and interference from environmental and food intrinsic factors	[25,26]
Array sensors	Simple, low cost and no need of chemical reagents, multi analysis	Unstable sensor due to environmental factors (temperature, humidity) and other gases in the air.	[27]
Microfluidic system	Simple, rapid, minimum sample consumption, multi-functional integration, small size, multiplex detection and portability	Disposable leading to high detection cost, require high technology (e.g. antibody immobilization), difficult integration of microfluidic chips and peripheral devices	[28, 29]
IoT, blockchain, and radio frequency identification (RFID)	Simple, decentralized data management, guaranteed data security, simultaneous data integration, wide application, and lower communication cost	Unable to eliminate the use of unauthorized or fraudulent foodstuffs itself	[30, 31, 32]
Smartphone-based analysis	Feasible, low cost, records organization, and practical	Still dependent or semi-dependent on laboratory	[33]

### 2.2. Biosensors in supporting smart food traceability

7In a similar direction with the development of smart food traceability, research surrounding biosensors has attracted researchers’ attention. In fact, the current development of biosensors has further surpassed the disadvantages of different portable traceability technologies. Biosensors are well-known in the food supply chain for meeting the critical demand for onsite and immediate diagnosis and treatment of food quality control. This is because biosensors enable rapid yet selective, sensitive, and cost-effective detection of targeted analytes. Its ability to be easy-to-use without the need for complicated and expensive sample preparation has been one of the key features to be applied to point-of-care (POC) technology [34]. POC in the food supply chain usually revolves around the concerns of nutrient monitoring, food safety and security, and food production environment control [35].

**Figure 2.**
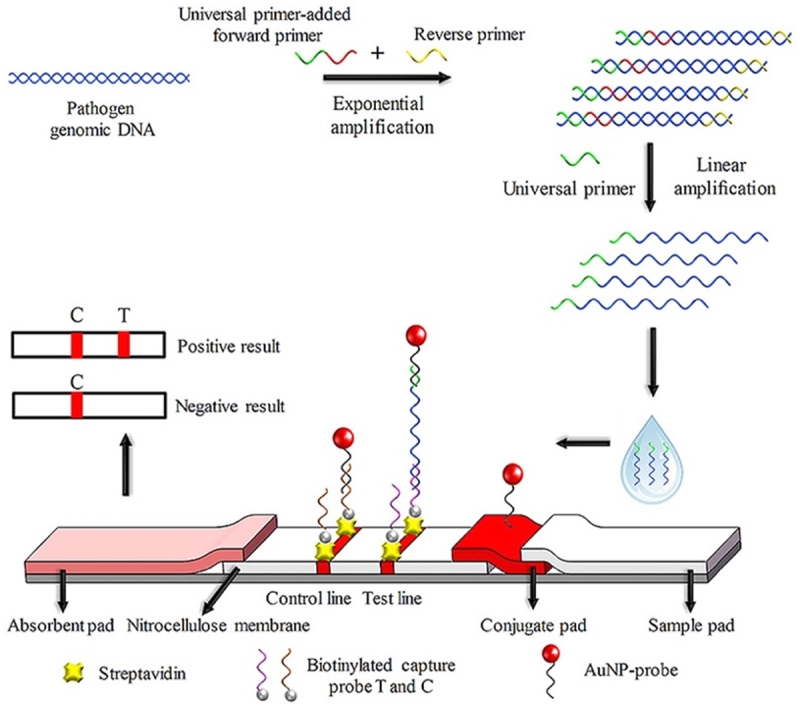
Mechanism of AuNP-based biosensor based on UP-APCR for rapid detection of *P. infestans*. A region of *P. infestans*-specific repetitive DNA sequence was amplified to generate large amounts of ssDNA using APCR. The ssDNA was then applied to the lateral flow biosensor, giving a characteristic red band when there is AuNPs accumulation. Reprinted from [36] with permission from Elsevier.

Within the pre-harvest stage, food crops might be exposed to microbial infestation due to aflatoxin contamination, deficiency of nutrients, extreme weather conditions (drought and floods), and others. Thus, early detection and routine control (i.e. traceability) are urgently needed to prevent pre-harvest loss and further contamination in the supply chain. Many of the biosensors developed have been focusing on the detection of crop pathogens. For instance, a gold nanoparticle (AuNP)-based lateral flow biosensor integrated with universal primer-mediated asymmetric polymerase chain reaction (UP-APCR) was developed for rapid visual detection of *Phytophthora infestans*, the casual late blight disease in potatoes and tomatoes [36]. The visual detection was done using sandwich-type hybridization assays with a detection limit of 0.1 pg/μL genomic DNA and high specificity within 1.5 hours. Figure 2 presents the mechanisms of the developed biosensor for rapid detection of *P. infestans* [36].

Other biosensors were developed to minimize abiotic stress-mediated crop loss based on phytohormone responses, the production of small molecules, free radicals, etc. An electrochemical biosensor to monitor phytohormones, such as salicylic acid, was developed by utilizing microneedle-based electrodes. The electrodes are known to be functionalized with a layer of salicylic acid-selective magnetic molecularly imprinted polymers. The biosensor showed a detection limit of 2.74 μM in both *in vitro* and *in vivo* [37]. More examples of biosensor application and comparison with conventional techniques within the pre-harvest stage (i.e. in the agriculture) are summarized in Table 2 as well as elaborately described elsewhere [58–60].

**Table 2.** Recent advancement of biosensors as smart food traceability system.

Category	Advancement	Advantage	Future direction	References
*Agriculture*	Monitor dissolved oxygen in water	In-situ, continuous, and autonomous	Stable over long-term performance	[38]
	Detect antibiotics in soil	Simultaneous, easily parallelizable, cost-effective	Specifically measure the concentration of a particular tetracycline type	[39]
	Monitor soil contamination	Simple, reliable, safe, inexpensive, portable, highly responsive, ambient light blocked, temperature controlled, and water jacketed	Real time application in soil	[40, 41]
	Detect plant infections, abiotic stress, metabolic content, phytohormones, miRNAs, genetically modified (GM) plants	On-site, in-vivo, online, and fast detection and reproducibility	More research and development	[42, 43]
*Food quality*	Determine polyphenols	Easy sample preparation, selective and sensitive, reproducible, low cost, portable, wide linear range, and accurate with excellent limit of detection (LOD)	Simple optimization method to limit interference of electrodeposition of nanoparticles	[44]
	Assess antioxidant capacities	Sensitive and precise, fast response time, and ease of miniaturization	Integration of intelligent devices, functional material application and model diversification, and explicit mechanism	[45]
	Assess food authenticity and detect illegal food additives	Highly selective and sensitive, facile, robust, portable, cost effective, higher detectability, universal	Modification of nanoparticles with specific ligands to improve selectivity, simple sample pretreatment	[46, 47]
	Detect food freshness	Highly sensitive, low cost, robust, and portable	Increase rate of reusability with simple cleaning process	[48]
	Quantify ethanol in beverages	Simple, fast, and highly sensible with elevated stability and biocompatibility	Usage of nanomaterials to enhance sensibility and applied for monitoring fermentation stage	[49]
	Monitor survival and freshness of fish	Simple, rapid, and accurate	Longer lifespan, stable over environmental factors, multiple freshness marker measured, and low cost	[50]
*Food safety*	Detect allergens	sensitive, selective, low-cost, and time-efficient	Associations of different transducer systems and nanomaterials with novel immobilization methods	[51]
	Detect antibiotics	Simple, low price, rapid response, real-time, good selectivity and sensitivity, easy miniaturization	Improvement in electrode materials (e.g. improve electrical conductivity and catalytic activity, amplifies biorecognition events), usage of different kind of nanomaterials, development of aptamers and molecularly imprinted polymers (MIPs) for multi-target analysis	[52], 53]
	Detect pathogenic microorganisms	Rapid, real-time, easy to carry out, and less labor-intensive	More sensitive and specific portable biosensor for utilization on farms to detect pathogens of fresh produce surface	[54]
	Detect fungal and bacterial toxins	High specific affinity, good chemical stability, low cost, easy to synthesis and modification	Sunlight powered and self-powered biosensor, split-type PEC biosensors and integrating PEC biosensing with arrays, microfluidics and chips for high-throughput and automation analysis	[55]
	Detect chemical contaminants (e.g. heavy metals, pesticides)	Low cost, continuous, specific, real-time, rapid, multiple analysis	Lower production cost to promote commercialization, modular assembly for real-time POC analysis, incorporation with nanotechnology and CRISPR-Cas-based diagnosis	[56, 57]

**Figure 3.**
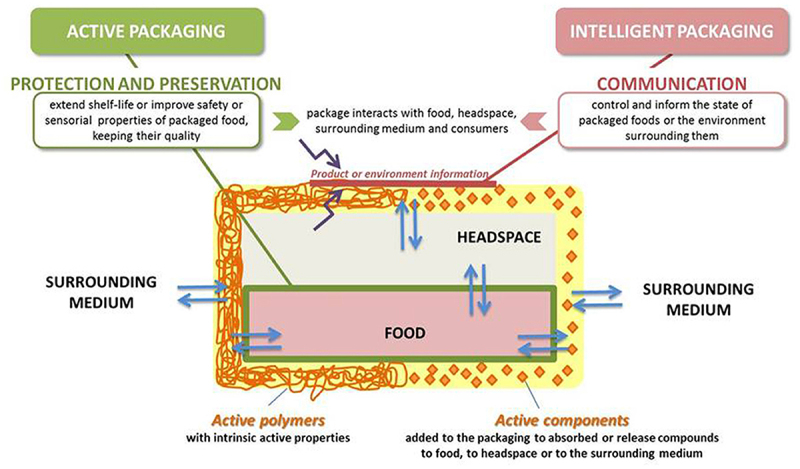
General schematic diagram of active and intelligent food packaging. Reprinted from [63] with permission from frontiers (CC-BY 4.0 license).

On the other side, the post-harvest stage usually consists of a more aggregate and complex process, including harvesting, sorting, storage, processing, packaging, distribution, and consumption. Application of biosensor in the post-harvest stage generally deals with food safety and authentication analysis which are mainly done during production and processing to ensure the food’s suitability. The analysis might include internal (e.g. nutrients, taste, pH, acidity, enzymes, etc.) and external (e.g. color, odor, texture, etc.) qualities [58]. As food is still constantly moved from one process to another until it is bought by consumers, the need for smart traceability is still urgent. This is because, after being packaged, the food is still prone to contamination and deterioration due to changes in the surrounding environment. While biosensors are known for their great ability to conduct onsite food safety and analysis, the recent development of biosensors as part of smart food packaging has further shown the great potency of biosensors in food traceability systems. As an active and intelligent system, smart food packaging enables manufacturers and consumers to trace the product’s conditions during storage and distribution while extending and maintaining the shelf-life and quality of the food [61,62]. This smart packaging has been incorporated into perishable products such as dairy, meat, seafood, fruits and vegetables, as well as bakery and confectionery products in recent years. The schematic mechanism of active and intelligent food packaging is shown in Figure 3 [63].

The underlying mechanism of active packaging to prolong shelf-life, maintain nutritional and organoleptic quality, inhibit microbial contamination or growth, and prevent the contaminants’ migration is known through the interaction of the product, package, and environment through the absorption of oxygen, ethylene, moisture, carbon dioxide (CO_2_), and odors, as well as the release of CO_2_, ethanol, flavor, and antimicrobial agents [61]. Meanwhile, intelligent packaging systems play roles to detect, record, trace, or communicate information about the food products within the food chain by perceiving information concerning the initial food composition and storage condition, headspace composition, and microbial growth through three principal systems: indicators, sensors, and radio frequency identification systems (i.e. data carriers) [64].

A biosensor incorporated with nanomaterials (i.e. nanosensors) has been extensively researched for their excellent prospects in food safety analysis and smart packaging. Nanomaterials have been explored for their great antimicrobial, mechanical, optical, and thermal properties to indicate the freshness, period for safe consumption, storage temperature, and others of food [65]. Current discoveries of the combined integration of biosensors with nanomaterials include the development of a microfluidic colorimetric biosensor using gold nanoparticles (AuNPs) for rapid detection of *Escherichia coli* O157:H7 concentrations in chicken samples with color change output [66]. This combined integrated biosensor and nanomaterials have shown a breakthrough in the challenges of smart food packaging, where gold nanoparticles provide an excellent platform for fast, low-cost, portable, and on-site food safety biosensors through their hydrogen bonding, nucleic acid hybridization, aptamer-target binding, antigen-antibody recognition, enzyme inhibition, and enzyme mimicking activity [67]. Another great bio-based material employed as biosensors for food packaging is chitosan-based hydrogels that have antimicrobial, antioxidant, and biodegradability qualities [67]. Table 2 summarizes the present development of biosensors in smart food traceability system from pre-harvest stage (i.e. agriculture) to post-harvest stage (i.e. to determine food quality and safety).

## 3. Biosensors in food safety and security

Increased food demand because of exponential population growth have prompted the need to frame the food security challenge and solution through food system transformation. The food system should adopt a multidimensional approach at all stages of the food supply chain (from production to consumption) to be environmentally, economically, and socially sustainable, resilient, and efficient [68,69]. As mentioned by [70], technologies such as remote sensing, tracing and tracking, active packaging, etc. are promising tools to tackle food security issues. This is because it could reduce the demand trajectory, fill the production gap, and avoid production losses. Biosensors represent a cutting-edge frontier in food traceability systems, enabling smart food safety and quality management tools. Thus, food security issues due to food contamination and the deterioration of nutrients and qualities could also be easily traced and prevented.

There have been many types of biosensors developed around food safety and quality tools, yet the main classification of biosensors in foodborne applications is based on their transducers. Optical biosensors, whose output signal is light emission, usually allow direct (label-free) detection of foodborne pathogens. The basic detection principle is usually found when cells bind to receptors or become immobilized on the transducer surface, causing changes that can be detected by the sensors. Electrochemical biosensor detection, on the other hand, is primarily relate to the ability to detect specific molecules (e.g. DNA-binding drugs, glucose, hybridized DNA). The principle is based on the measurable electrons or ions that are produced or suppressed by different types of chemical reactions [71].

The applications of biosensors within food safety and security include the detection of foodborne pathogens, toxins, veterinary drugs, pesticides, and other chemical contaminants (i.e. food allergen, heavy metals, etc.) as described next. Table 3 summarizes the development of biosensors to detect contaminants that concern food safety and quality.

**Table 3.** Recent development of biosensors in food safety and quality field.

Category	Analyte	Type	Bioreceptor	Food sample	LOD	Linear range	References
Pathogen	*Salmonella* sp.	Magnetic	Phage	Orange juice	5 CFU/mL	10^2^ to 10^8^ CFU/mL	[72]
	*Salmonella* sp.	Electrochemical	Phage	Chicken	1.3 × 10^2^ CFU/mL	2 × 10^2^ to 2 × 10^5^ CFU/mL	[73]
	*Salmonella* sp.	Calorimetric	Aptamer	Fresh-cut vegetable	6.0 × 10^1^ CFU/mL	6.0 × 10^1^ to 6.0 × 10^5^ CFU/mL	[74]
	*Salmonella typhimurium*	Electrochemical	DNA	Egg, milk	1 CFU/mL	1.8 × 10^5^ to 1.8 CFU/mL	[75]
	*Campylobacter jejuni*	Fluorescence	Antibody	Poultry liver	10 CFU/mL	10 to 10^6^ CFU/mL	[76]
	*Campylobacter jejuni*	Fluorescence	Aptamer	Livestock and dairy	3 CFU/mL	10 to 10^7^ CFU/mL	[77]
	*E*. *coli* O157:H7	Chemiluminescence	DNA	-	130 CFU/mL	2 × 10^2^ to 10^8^ CFU/mL	[78]
	*E*. *coli* O157:H7	Electrochemical	Phage	Fresh milk and raw pork	11.8 CFU/mL	10^2^ to 10^7^ CFU/mL	[79]
	*E. coli*	Magnetic	Aptamer	-	1 × 10^2^ CFU	100 to 400 μg/mL	[80]
	*Yersinia enterocolitica*	Single walled carbon nanotube	Antibody	Kimchi	10^4^ CFU/mL	10^6^ to 10^4^ CFU/mL	[81]
	*Vibrioparahaemolyticus*	Electrochemiluminescence	Aptamer	-	1 CFU/mL	1 to 10^6^ CFU/mL	[82]
	*Shigella flexneri*	Electrochemical	DNA	-	7.4 × 10^−22^ mol/L	8 × 10^10^ to 80 cells/ml	[83]
	*Staphylococcus aureus*	Fluorescence	Aptamer	Pork and beef	25 CFU/mL	63 to 6.3 × 10^6^ CFU/mL	[84]
	*Vibrio cholerae*	Electrochemical	DNA	-	7.41 × 10^−30^ mol/L	10^−8^ to 10^−14^ and 10^−14^ to 10^−27^ mol/L	[85]
	Norovirus	Electrochemical	Antibody	-	60 ag/mL	1 fg/mL to 1 ng/mL	[86]
	Rotavirus	Electrochemical	Phage	-	5 copies/mL	10^1^ to 10^5^ copies/mL	[87]
Veterinary drug	Ampicillin	Optical	Antibody	Milk	7.4 × 10^−10^ g/mL	4 × 10^−5^ to 4 × 10^−9^ g/mL	[88]
	Ampicillin	Electrochemical	Aptamer	-	1.33 fg/mL	1.0 × 10^−5^ to 5.0 ng/mL	[89]
	Penicillin sodium	Electrochemical	Enzyme	Milk	0.64 ng/mL	0.1 to 10 ng/mL	[90]
	Kanamycin	Electrochemical	Enzyme	-	0.5 pM	1 pM to 1 μM	[91]
	Oxytetracycline	Electrochemical	Aptamer	Milk	30.0 pM	1.00 to 540 nM	[92]
	Oxytetracycline	Electrochemical	Antibody	-	0.33 ng/mL	1 to 200 ng/mL	[93]
	Tetracycline	Electrochemical	Aptamer	Milk	3 × 10^−17^ M	1 × 10^−16^ to 1 × 10^−6^ M	[94]
	Sulfameter	Fluorescence	Aptamer	-	1.57 ng/mL	2 to 250 ng/mL	[95]
Mycotoxin	Aflatoxin B1	Electrochemical	Aptamer	Wine and soy sauce	0.016 pg/mL	0.1 to 10 pg/mL	[96]
	Aflatoxin B1	Electrochemical	Antibody	Corn	0.54 pg/mL	1 pg/mL to 10 µg/mL	[97]
	Aflatoxin B1	Fluorescence	DNA	Peanut	0.92 pg/mL	0.001 to 80 ng/mL	[98]
	Ochratoxin A	Optical	Enzyme	Maize	54 pg/mL	0.1 to 50 ng/mL	[99]
	Ochratoxin A	Electrochemiluminescence	Enzyme	-	3 pg/mL	0.01 to 5 ng/mL and 5 to 100 ng/mL.	[100]
	Ochratoxin A	Fluorescence	Aptamer	Rice	0.005 ng/mL	0.01 to 10 ng/mL	[101]
	Ochratoxin A	Fluorescence	Aptamer	-	0.36 nmol/L	0.69 to 8.0 nmol/L	[102]
Pesticide	Carbendazim	Fluorescence	Aptamer	-	0.05 ng/mL	0.1 to 5000 ng/mL	[103]
	Carbaryl	Colorimetric	Enzyme	-	0.008 ng/mL	0.01 to 0.25 ng/mL	[104]
	Carbaryl	Electrochemical	Enzyme	Apple	4.5 nmol/L	5.0 to 30.0 nmol/L	[105]
Food allergen	Ara h1	Fluorescence	Aptamer	-	0.04 ng/mL	0.1 to 100 ng/mL	[106]
	Ara h1	Electrochemical	Aptamer	Cookie dough	21.6 ng/mL	50 to 1000 ng/mL	[107]
	Tropomyosin	Magnetic	Aptamer	Seafood	30.76 ng/mL	0.1 to 2.5 μg/mL	[108]
	Tropomyosin	Fluorescence	Antibody	Fish fillet and meatball	0.01 μg/mL	0.005 to 1 μg/mL	[109]
	Ovomucoid	Electrochemical	Phage	-	0.12 μg/mL	1.55 to 12.38 μg/mL	[110]
	Arginine kinase	Fluorescence	Aptamer	Shellfish	0.298 μg/mL	0 to 2.5 μg/mL	[111]
	Beta-lactoglobulin	Fluorescence	Aptamer	-	0.048 mg/L	0.39 to 1000 mg/L	[112]
	Beta-lactoglobulin	Fluorescence	Aptamer	Infant food products	96.91 μg/L	0.36 to 500 mg/L	[113]
Heavy metal	Mercury	Colorimetric	Whole-cell	-	0.1 ppm	0.1 to 0.75 ppm	[114]
	Cadmium				0.2 ppm	0.2 to 0.75 ppm	
	Copper				2 ppm	2 to 7.5 ppm	
	Lead (II)	Electrochemiluminescence	Aptamer	Water	0.059 ng/L	0.1 to 1 × 10^6^ ng/L	[115]
	Arsenic (III)	Electrochemical	Whole-cell	Water	1.5 ppb	2.5 to 50 ppb	[116]
	Copper (II)	Bioluminescence	Enzyme	Water	2.5 mg/L	-	[117]
Others	Acrylamide	Fluorescence	DNA	Bread crust	2.41 × 10^−8^ M	5 × 10^−3^ to 1 × 10^−7^ M	[118]
	Hypoxanthine	Colorimetric	Enzyme	Fish	8.22 μmol/L	0.01 to 0.16 mmol/L	[119]
	Hypoxanthine	Electrochemical	Enzyme	Fish	15 μM	50 to 800 μM	[120]
	Xanthine	Electrochemical	Enzyme	Fish	0.35 nM	0.001 to 0.004 μM and 0.005 to 50.0 μM	[121]

### 3.1. Detection of foodborne pathogens

The majority of foodborne disease outbreaks are caused by various forms of pathogenic bacteria, viruses, and parasites. Among the severe and fatal bacterial infections, *Salmonella*, *Campylobacter*, and enterohaemorrhagic *E. coli* are the most common pathogens, affecting millions of people annually. The symptoms might include fever, headache, nausea, vomiting, abdominal pain, and diarrhea. Outbreaks of salmonellosis are usually linked with eggs, poultry, and other products of animal origin, while foodborne cases caused by *Campylobacter* are mainly caused by raw milk, raw or undercooked poultry, and drinking water. Enterohaemorrhagic *E. coli*, on the other hand, is usually associated with unpasteurized milk, undercooked meat, and contaminated fresh fruits and vegetables. Other bacteria that have caused foodborne diseases are *Listeria* infections from unpasteurized dairy products and various ready-to-eat foods, and *V. cholerae*, which mainly contaminate rice, vegetables, millet gruel, and various types of seafood [4].

**Figure 4.**
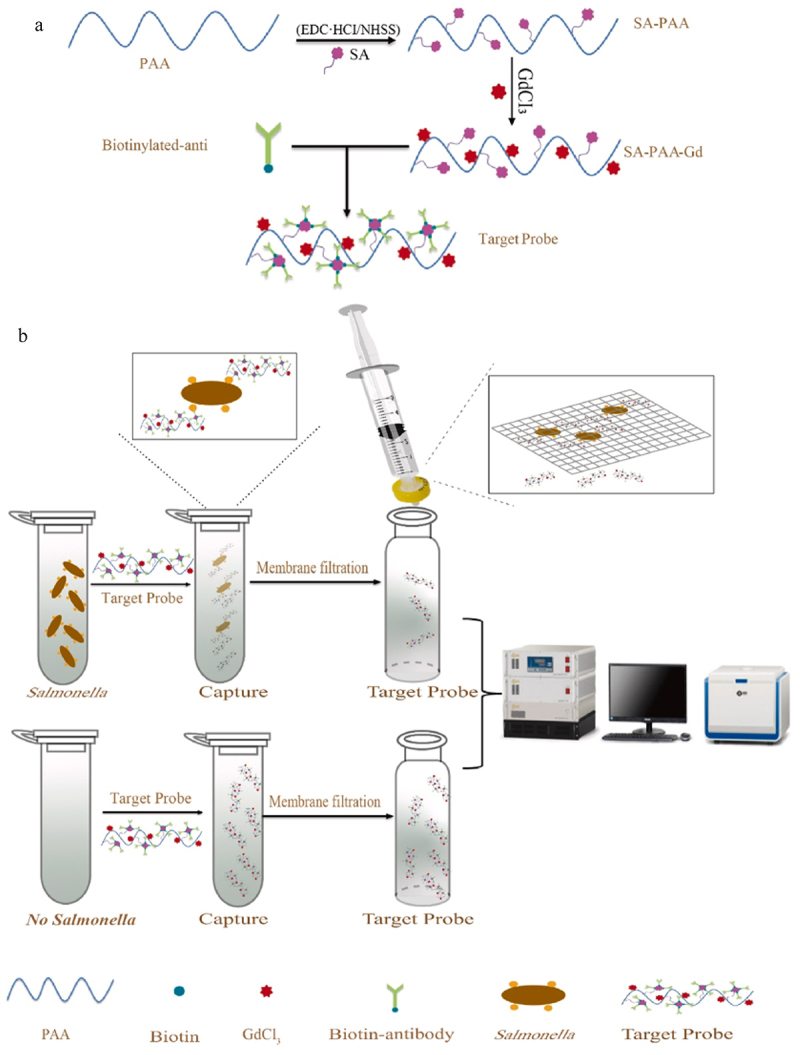
Schematic diagram of NMR biosensor to detect *salmonella* in milk. Firstly, the target probe was prepared (a) followed with detection of *salmonella* in milk (b). Reprinted from [123] with permission from Elsevier.

Recently, a ratiometric electrochemical biosensor based on the combination of SRCA (Saltatory Rolling Circle Amplification) and the CRISPR/Cas12a (CRISPR associated with system 12a) system for ultrasensitive and specific detection of *Salmonella* in food was developed. The basic principle of detection lies in the self-calibration of ratiometric electrochemical measurement to reduce internal or external disturbances, along with specific signal amplification using rapid SRCA amplification technology and the *trans*-cleavage capabilities of Cas12a. The biosensor displayed a detection limit as low as 2.08 fg/μL of *Salmonella* in pure culture and 100% sensitivity, 97.8% specificity, and 98% accuracy in the actual sample [122]. Another low-field NMR (Nuclear Magnetic Resonance) biosensor based on a high-density carboxyl polyacrylate targeting gadolinium (Gd) probe was developed to rapidly detect *Salmonella* in milk. Figure 4 presents the schematic diagram of the principle of the NMR biosensor for detecting *Salmonella* in milk samples. At first, the target probe was obtained through an amide reaction resulting in activated polyacrylic acid and streptavidinylated polyacrylic acid (SA-PAA), which further undergoes a chelating adsorption reaction for gadolinium. The target probe of SA-PAA-Gd was then used to capture *Salmonella* through antigen-antibody interaction. This biosensor has shown a detection limit of 3.3 × 10^3^ CFU/mL within 1.5 hours [123].

*E*. *coli* O157:H7, as part of the Shiga-toxin-producing *E. coli* (STEC), and *C. jejuni* infections have also presented an alarming challenge in food safety. [124] developed a microfluidic chemiluminescence biosensor based on multiple signal amplification of a combined CHA with H2-Au NP-catalyzed CL reaction for rapid and ultrasensitive detection of *E. coli* O157:H7. A label-free, specific, rapid, and cost-effective electrochemical biosensor has also been successfully developed using phage EP01 as the recognition agent for detection of *E. coli* O157:H7 GXEC-N07 in fresh milk and raw pork [125]. For *Campylobacter* detection, a whole-cell *V. harveyi*-based biosensor assay developed to accurately quantify and observe the interspecies signaling molecule of *C. jejuni* called autoinducer-2 (AI-2) has shown great prospect in complex food matrices of food production [126]. A paper-based DNA biosensor based on an enhanced chemiluminescence signal on a DNA dot blot and a silica nanoparticle was also developed to monitor *Campylobacter* in naturally contaminated chicken meat without pre-amplification [127].

**Figure 5.**
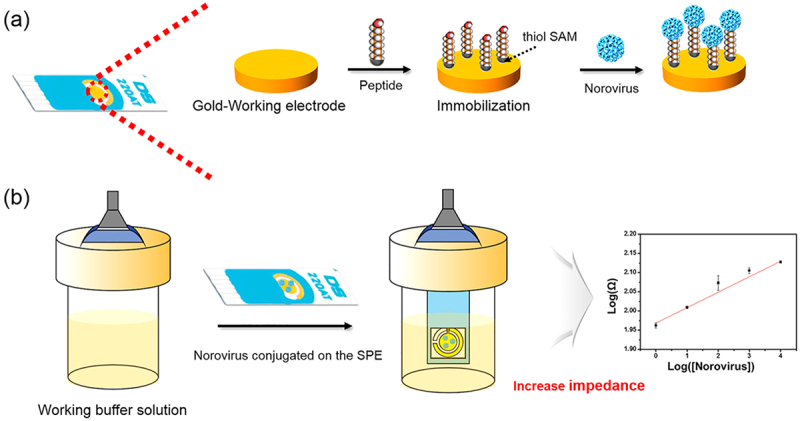
Detection of Norovirus using peptide-coated electrochemical biosensor. Reprinted from [129] with permission from Elsevier.

In viral pathogens, norovirus (NoV) is one of the most common foodborne infections, causing nausea, explosive vomiting, watery diarrhea, and abdominal pain [4]. Given that NoV causes over 200,000 deaths each year, [128] created a photoelectrochemical biosensor coupled with a novel custom-made monoclonal antibody as a convenient POC system for diagnosing NoV infection and detecting NoV-contaminated food samples. An electrochemical biosensor based on specific binding peptides coated onto the gold electrode has also exhibited highly specific detection of NoV from oysters [129]. The schematic illustration of the biosensor to detect NoV is shown in Figure 5 [129]. Similarly, [130] also developed a 3D electrochemical aptasensor for NoV detection in spiked oysters based on phosphorene-gold nanocomposites.

### 3.2. Detection of mycotoxins

Mycotoxins, the secondary metabolites produced by fungi, have been known to have a significant toxicity effect on human and animal health, economies, and international trade. Mycotoxin contamination might occur on crops either during harvesting or storage. There have been about 300 mycotoxins identified and reported to contaminate 30 to 100% of food and feed samples in the world. Five major mycotoxin groups that are commonly found are aflatoxins (AF), ochratoxins (OTA), fumonisins, zearalenone (ZEN), and deoxynivalenol/nivalenol (DON) [131]. As multiple mycotoxin contamination in foodstuffs poses synergistic effects that cause a more significant threat to human health, portable chemiluminescence optical fiber aptamer-based biosensors for ultrasensitive onsite assay of multiplex mycotoxins in food are developed [132]). With a LOD of 0.015–0.423 pg/mL, the biosensor demonstrated sensitive and multiple analysis of mycotoxins in infant cereals. The selective and multiple analysis of the biosensor is mainly based on optical fibers that have specific recognition of single-stranded binding proteins (SSB) and mycotoxin aptamers (Jia et al., 2022).

**Figure 6.**
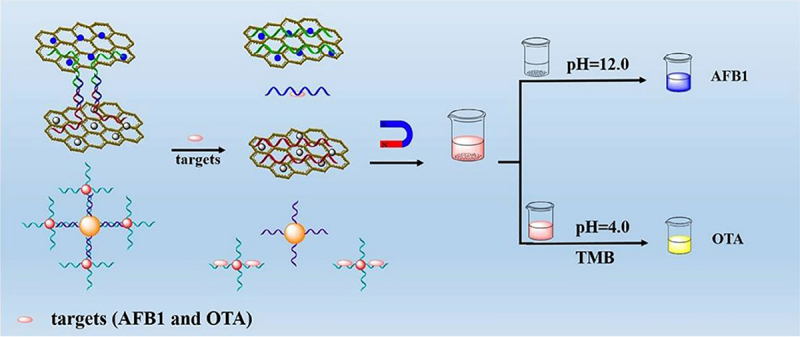
Detection principle of a colorimetric biosensor to detect AFB1 and OTA. Formerly, the two platforms of Fe_3_O_4_/GO and TP-GO and Fe_3_O_4_@Au and Au NPs were formed through the combination of an AFB1 aptamer and the complementary strands of an OTA aptamer and probe, respectively. The absence and presence of both AFB1 and OTA will result in platform separation and a colorless supernatant. The addition of an alkaline solution to magnetically separated solids and the usage of Au NPs in the supernatant, on the other hand, results in a dark blue-colored solution. Reprinted from [134] with permission from Elsevier.

Another portable chemiluminescence biosensor for rapid on-field screening and quantification of OTA in wine and coffee samples was developed [133]. The user-friendly smartphone-based biosensor was developed using the combination of low-cost, disposable analytical cartridges that contain a lateral flow immunoassay (LFIA) strip with the chemiluminescence detection system of the smartphone camera as a light detector. The biosensor showed a LOD of 0.3 and 0.1 μg/L for wine and coffee, respectively. On the other hand, [6] successfully developed an electrochemical biosensor based on *E. coli* as the signal recognition element, *p*-benzoquinone as the mediator, and a two-step reaction procedure. The biosensor showed detection limits of 1 and 6 ng/mL for Aflatoxin B1 (AFB1) and ZEN, enabling a promising tool for toxicity evaluation in corn and peanut oils. Moreover, a colorimetric biosensor with a wide detection range for dual mycotoxins detection was developed using a Fe_3_O_4_/GO-based platform for AFB1 detection and a Fe_3_O_4_@Au based platform for OTA detection [134]. The detection principle of a colorimetric biosensor is shown in Figure 6 [134].

### 3.3. Detection of veterinary drug

**Figure 7.**
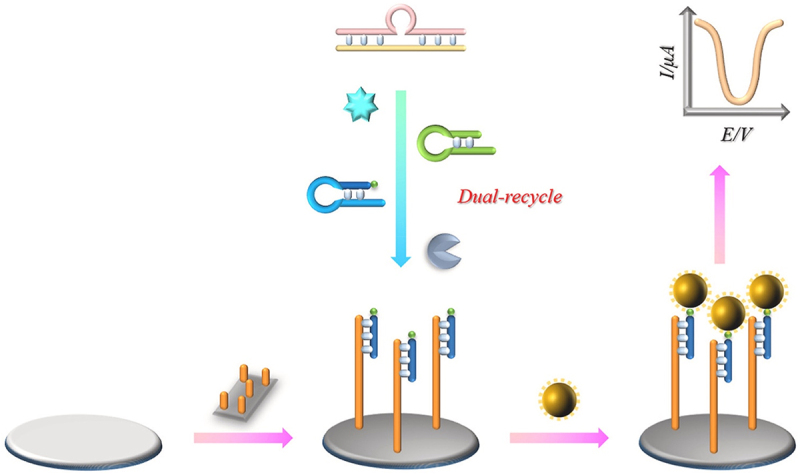
Schematic diagram of electrochemical biosensor based on exonuclease-III-assisted dual-recycling amplification for rapid, sensitive, and accurate detection of kanamycin in milk. Reprinted from [136] with permission from Elsevier.

Antibiotics, such as chloramphenicol, sulfadiazine, neomycin, and kanamycin, are the major group of veterinary drugs used in food-producing animals to prevent or cure disease. The misuse of veterinary drugs (i.e. antibiotics) often leads to the deposition of drug residues in the tissues and organs of food animals. This will then induce serious health hazards (e.g. allergies, antimicrobial resistance) when accumulated in the human body. [109] have successfully fabricated an ultrasensitive label-free biosensor based on aptamer-functionalized 2D photonic crystal (SiO2-Au-ssDNA 2D PC) to detect kanamycin in milk. With the combination of the negatively charged AuNPs and sulfhydryl-modified ssDNA, the biosensor has resulted in excellent performance with a LOD of 1.10 pg/mL [135]. A more recent development of an electrochemical biosensor with a LOD of 0.6 pM to detect kanamycin in milk was also manufactured based on exonuclease III-assisted dual-recycling amplification [136]. The ultrasensitive and catalytic signal amplification of the biosensor were constructed using high-conductive MXene/VS_2_ and high-activity CeCu_2_O_4_ bimetallic nanoparticles as the electrode surface and nanozyme, respectively. Meanwhile, the accurate detection of the biosensor was fabricated from the dual supplementary recycling of primer DNA and hairpin DNA (Figure 7).

Another ultrasensitive and selective colorimetric biosensor based on G-quadruplex DNAzyme was also developed to detect residues of tetracyclines in foods [137]. Tetracycline antibiotics (e.g. tetracycline (TET), oxytetracycline (OTC), chlortetracycline (CTC), and doxycycline (DOX)) are widely used in the field of livestock husbandry, and when they are misused, their residues are often found in animal-derived foods such as milk, honey, and pork [138]. The buildup in the human body can lead to serious diseases such as liver damage, tooth yellowing, allergic disorders, intestinal flora disorders, and bacterial resistance. The underlying mechanism of the biosensor to give results of tetracycline detection that could be determined even by the naked eye is based on the reaction between tetracycline and DNAzyme, which is composed of hemin and G-quadruplex and has peroxidase-like activity to form a stable complex and reduce catalytic activity. This reaction will then cause the solution’s color to change from yellow to green [137]. A more recent similar colorimetric biosensor to detect tetracycline antibiotics was constructed with a LOD of 0.333 ng/mL [139].

Furthermore, ampicillin (AMP), as one of the most used β-lactam antibiotics with antibacterial activity against gram-negative and positive bacteria, is also extensively used in agriculture, livestock, poultry, aquaculture, etc. There have been a number of severe environmental and food safety concerns recorded due to the overdose of this antibiotic, including endocarditis, membranitis, intestinal infection, and irritability. Yadav et al. [140] have successfully fabricated a label-free electrochemical immunosensor based on molybdenum disulfide nanoparticles modified disposable indium tin oxide (ITO) with a LOD of 0.028 µg/mL in different food samples (milk, orange juice, and tap water). Detection of sulfamethazine, which is the most widely used and detected sulfonamide in animal-derived foods, was also studied with an antibody-antigen-aptamer sandwich electrochemical biosensor [141].

### 3.4. Detection of pesticides

**Figure 8.**
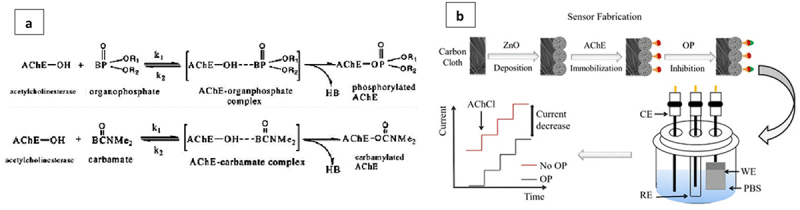
(a) inhibition activity of AChE by OP and carbamate pesticides. Reprinted from [146] with permission from hibiscus Publisher (CC by 4.0 license); (b) schematic diagram of zinc oxide (ZnO)-based biosensor to detect OP. Reprinted from [147] with permission from MDPI (CC by 4.0 license).

Organophosphate (OPP) and carbamate pesticides have had a positive impact on insect pest control and crop production globally. However, the indiscriminate and widespread use may result in impending toxicity to the environment and human health [142]. The progressive research on the development of various biosensors has ranged from the use of conventional immobilizing supports to more advanced hybrid or composite nanomaterials [143]. Previous reviews by [144] and [145] have summarized different enzymatic electrochemical biosensors for pesticide detection in foods. In particular, the inhibition-based biosensors that utilize the acetylcholinesterase (AChE) enzyme are shown to be mostly preferred. This is because the toxicity of organophosphorus pesticides will result in the formation of covalent bonding and the permanent inactivity of the AChE enzyme (Figure 8a).

For instance, a low cost and highly sensitive biosensor which immobilized the AChE enzyme on zinc oxide (ZnO) demonstrated excellent performance with the detection limit range from 0.5 nM–5 µM [147] (Figure 8b). A graphene/chitosan/parathion multi-residue electrochemical biosensor was also fabricated to detect 11 types of OP pesticides through an indirect competitive method [148]. The biosensor was prepared by combining the formation of phosphorylated AChE between organophosphorus molecules and AChE as well as the excellent conductivity of graphene. Likewise, a portable electrochemical biosensor was constructed by integrating a laser-induced graphene (LIG) electrode on polyimide (PI) foil and MnO_2_ nanosheets loaded on the paper [149]. With a ‘sign-on’ electrochemical response of OPs determination in vegetables, the detection principle of the biosensor is known to rely on AChE-catalyzed hydrolytic product-triggered disintegration of MnO_2_ nanosheets. Another type of biosensor, which is voltametric with confirmed reusability after 90 days, was also executed based on enzyme activity inhibition of fungal laccase and bacterial catalase [150].

### 3.5. Detection of other contaminants

Although it is not a common food allergen, there has been a growing incidence of mustard allergies. Therefore, a disposable electrochemical PCR-free biosensor was generated for the selective detection of protein Sin a 1, the most potent allergen in yellow mustard [151]. The detection principle was done through the formation of DNA/RNA heterohybrid-specific antibodies by sandwich hybridization, resulting in simple and fast detection with a LOD of 3 pM. In a similar direction, [152] have successfully fabricated an aptameric biosensor using graphene oxide to detect the alarming shrimp allergy due to tropomyosin. The advancement of biosensors that allow allergen detection and evaluation of allergy drugs was also studied. Jeong et al. [153] constructed a bioelectronic sensor based on nanovesicles combined with anti-immunoglobulin E (anti-IgE) antibody receptors for signal amplification. The sensing system showed that it was sensitive and selectively able to detect the peanut allergen *Arachis hypogaea* 2 (Ara h 2) *with a* LOD of 0.1 fM in real food samples such as peanut and egg white.

**Figure 9.**
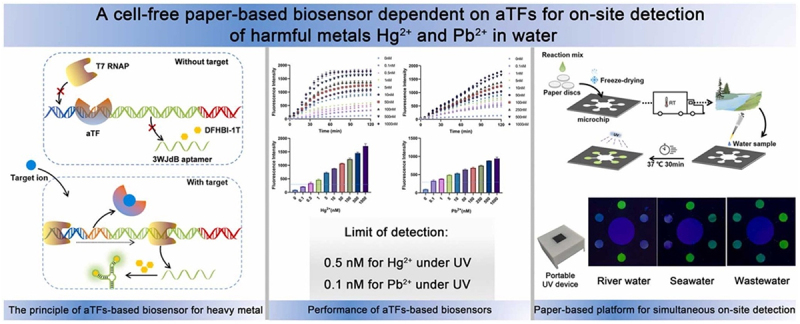
Schematic diagram of a cell-free paper-based biosensor for on-site detection of heavy metals Hg^2+^ and Pb^2+^ in water based on aTfs. Reprinted from [154] with permission from Elsevier.

Heavy metals (e.g. mercury, lead, cadmium, and arsenic) are known to pose a serious threat to food safety if consumed above the weekly allowable intake. [154] developed a cell-free paper-based biosensor for on-site detection of Hg^2+^ and Pb^2+^ in water using a combination of *in vitro* transcription (IVT) technology with allosteric transcription factors (aTFs). The detection principle mainly relied on the aTFs specific affinity characteristic toward metal ions that cause dissociation from DNA and result in a measurable signal of transcribed fluorescent RNA (Figure 9). Copper is a heavy metal that is also classified as an essential micronutrient for performing various bodily functions for plant, animal, and human health (e.g. production of red blood cells, collagen, energy, etc.). Copper should be monitored on a regular basis to avoid toxicity and health problems caused by over- and underconsumption [155]. Žunar et al. [156] have successfully transformed the native copper response of yeast *S. cerevisiae* into a whole-cell eukaryotic whole-cell copper biosensor to evaluate copper bioavailability.

Food additives, in particular hydrogen peroxide (H_2_O_2_), are strong oxidizing agents that are often used in food processing as a bleaching agent in wheat flour, an antimicrobial agent in milk, or a sterilizing agent for food packaging materials. As high ingestion of hydrogen peroxide could result in significant health hazards, Vasconcelos et al. [157] developed a chemiluminescence biosensor using a hydroxyethylcellulose-based membrane to detect hydrogen peroxide (H_2_O_2_) in different types of milk (i.e. fresh-raw, whole, semi-skimmed, and skimmed milk). The biosensor proved to be a quick, environmentally friendly, and low-cost method for detecting H_2_O_2_ in milk, with a LOD of 1.0 × 10^−3^ % w/w for fresh, raw, skim, and whole milk, and 2.0 × 10^−3^ % w/w for semi-skimmed milk. Moreover, synthetic colorants that are often used to enhance the sensory properties of foods are known to contain azo compounds, which pose hazards to human health. A study by Manjunatha [158] developed a sensitive and selective cyclic voltammetric sensing system that utilized a poly (glycine) modified carbon paste electrode to determine tartrazine with a LOD of 2.83 × 10^−7^ mol/L.

## 4. Challenges and future perspective

Despite the necessity of traceability system within the complex food supply chain, not all food companies have sufficient economic value or scale to invest in. It is claimed by many studies that biosensors are cost-effective, yet the cost to manufacture biosensors still needs to be reduced as commercial applications are still lacking. Currently, self-powered biosensors based on biofuel cells have attracted great interest as they could advance the cost-efficiency of biosensors to another extent while being user-friendly and highly suitable for miniaturization, portability, and wearability [159]. Lack of uniformity in the systems, coordination, allocation of costs and benefits for research and development, as well as globalization pace, climate, geographical location, and natural resources between each country have pose further challenges for implementing efficient application of biosensor for food system. [160] have successfully developed a highly thermal and storage stable electrochemical biosensor for facilitating rapid pesticide detection of fruits and vegetables in a variety of climates. Another biosensor developed with long shelf life after 40 days also further supported the advancement of biosensor to tackle challenges in food safety and analysis technologies [161].

Furthermore, as nanomaterials could be toxic, the fabrication of biosensor using this material might rise other challenges related to health. Therefore, further study on green synthesis and incorporation of biocompatible materials have grasped the insurgencies along with enhancing the sustainability value of biosensors in the food system (i.e. repurpose, reuse, degradable, or recyclable material) [162]. Development of biosensors using microorganisms as the bioreceptor also have attracted many researchers’ attention. This is because the regulatory genes and proteins of microorganisms possess various responsive mechanisms to cope with environmental stress, pollutants, and heavy metals [163,164]. This then could be useful for the development of biosensor in agriculture as well as environmental monitoring. By having minimum requirements of electricity, water, gas, and energy from biosensor, minimal generation of carbon footprints could also be achieved.

As food system has become more complex, it is also urgently needed nowadays to have a more integrated food detection system. Biosensor combined with emerging technologies, such as smartphones, 3D printing, IoT, AI, and blockchain, could lead biosensor into another extend of advancement. For instance, combining biosensor and smartphone could significantly improve detection accuracy and shorten the detection time due to the automation and cloud-data saving from smartphone. With the current globalization where smartphones are being used by almost all people, smartphone-assisted biosensors will give enormous potential for onsite detection of food contaminants. For instance, Abdelbasset et al. [165] discussed that smartphone-based aptasensor offers a semi-automated user interface that can be exploited by an inexpert person, along with fast and wireless data transferability. It could then be a breaking stone for onsite, portable, and simple monitoring in the smart food traceability system. As for sensor array, it can improve the specificity of biosensor due to its ability to accurately identify very similar and wide range of analytes in mixtures for fingerprint identification. This then can be used for detecting any food adulteration. Lastly, biosensor and IoT technologies could lead in the wireless transmission technology [154,166].

## 5. Conclusion

The increased prevalence of foodborne illness and food insecurity have shown great urgency in developing smart food traceability systems that are rapid, accurate, reliable, low cost, and able to conduct multiple analyses. Many advancements in biosensors over the years have shown great promise in enabling whole process monitoring to tackle the uncertainty and complexity of the food supply chain. The present review highlights different fabrications of biosensors within the pre- and post-harvest stages of food (e.g. agriculture, detecting biological and chemical food contaminants, smart food packaging). Based on the current trends, many biosensors associated with nanoparticles have more advantages, such as a lower detection limit, higher sensitivity, selectivity, and stability over long-term usage. However, there are still, many challenges to be tackled and improved in the future.
